# Rapid Scoping Review on the Topic of Ensuring Social Protection and Basic Services to Inform the United Nations Framework for the Immediate Socioeconomic Response to COVID-19

**DOI:** 10.1177/00207314211024896

**Published:** 2021-06-07

**Authors:** Megan Highet

**Affiliations:** 1 CIHR Health System Impact Fellowship Alumni, Calgary, Alberta, Canada

**Keywords:** COVID-19, pandemic, social protection, basic services, equity, implementation science, sustainability, shock event, pandemic recovery

## Abstract

This rapid scoping review has informed the development of the UN Research Roadmap for the COVID-19 Recovery on the topic of “Ensuring Social Protection and Basic Services.” The aim was to provide a robust synthesis of key concepts and existing evidence drawn from a wide range of disciplines to support the identification and appraisal of research priorities. An emergent theme has been the notion that measures implemented in response to COVID-19 merely ameliorate symptoms of entrenched, systemic gender-, age-, and race-based inequity, inequality, and exclusion. Key findings include the critical role of contextual and community-based knowledge for informing the design, development, and delivery of programs, as well as the urgent need for implementation science to move existing knowledge into action. This review also describes how the disruption associated with “shock events” such as the COVID-19 pandemic is often associated with unusually high levels of interest and willingness to invest in programs and policies to strengthen strained systems. As such, an unprecedented window of opportunity exists to leverage measures implemented in response to the COVID-19 pandemic to effect large-scale, sustainable change and thereby increase the resiliency of our interconnected systems for the future.

This rapid scoping review has informed the development of the UN Research Roadmap for the COVID-19 Recovery on the topic of “Ensuring Social Protection and Basic Services.” Its purpose was to determine what is known about the effectiveness of interventions that have aimed to enhance social protection and/or ensure continuity of access to basic services during examples of previous shock events. A secondary objective was to assess the nature of any gaps in existing knowledge, which may point to research priorities for supporting action throughout the COVID-19 recovery period. While this rapid scoping review was not intended to be a systematic review of the literature, it was nevertheless necessary to ensure that a wide breadth of cross-disciplinary research was captured to inform a robust synthesis and support evidence-informed decision making.

Social protection is defined in the literature in many ways. For the purposes of this report, it is defined as “public actions taken in response to levels of vulnerability, risk, and deprivation which are deemed socially unacceptable within a given polity or society.”^1^^(p7)^ Many social protection measures are based on efforts to reduce chronic systemic poverty,^1^ but some are also intended to guard against large numbers of individuals and households falling below the poverty line, particularly during a shock event.^2,3^ A wide range of interventions fall under the umbrella of social protection, including cash or in-kind transfers, voucher programs, subsidies, and school feeding/nutrition programs.^2^ Regardless of the mechanism or target of social protection programs, the intent is always “to promote dynamic, cohesive, and stable societies through increased equity and security.”^1^^(p7)^ A common goal is often to reduce harmful coping strategies, such as selling off resources to meet immediate needs or pulling children out of school to contribute wages to the household economy, both of which leave families further disadvantaged in the future.^4^

The COVID-19 pandemic has caused tremendous hardships, particularly for vulnerable groups around the world. Although it is not the only significant infectious disease outbreak to have occurred in recent years, the COVID-19 pandemic is unprecedented in magnitude, meaning that no event that came before has resulted in such an intensive or widespread need for social protection responses.^5^ The evidence synthesized in this report should therefore be read with this in mind; nothing in recent history is comparable to the shock created by COVID-19, which to some extent limits the ability to generalize from historic examples.

## Methodology

The approach for completing this rapid scoping review was designed to balance the project's short timeline for completion (∼3 weeks) against a rigorous search and reporting methodology. The need for rapidly synthesizing evidence to inform decision making is a common challenge in the field of public health; however, best practice for conducting rapid scoping reviews is available to guide the work of researchers in this area. This rapid scoping review has therefore been structured around guidance offered by the National Collaborating Centre for Methods and Tools (see: https://www.nccmt.ca/tools/rapid-review-guidebook).

The field of social protection and basic services is enormous, and so the body of literature is vast.^1^ Considering the fact that this rapid scoping review was intended to include a broad scope of literature representing a diverse range of disciplines and areas of practice, it was necessary to narrowly focus the search criteria. To begin, the search was limited to focus on evidence from either the current or previous shock events. This decision was made because social protection programs in nonshock times leverage existing infrastructure and social networks to build resiliency.^5^ In times of shock, however, not only are the immediate needs to be addressed through social protection measures different than in times of stability, but the networks that would normally be relied upon to deliver social protection measures have very often been disrupted^6^ and therefore require different approaches for implementation.

The need to tailor in this way was made clear by another scoping review on the topic of social protection for the COVID-19 *response* period that was recently completed by Hebbar and Phelps.^5^ That review called for intensifying social protection measures in response to COVID-19 and explored logistical considerations for delivering a variety of cash transfer, school feeding, and public works programs. It ends with a recommendation for continued action and a call to intentionally build on social protection programs that were put in place during the response period to support scale, spread, and building of long-term resiliency.^5^

In the social protection literature, shock events are most commonly discussed as periods associated with natural disasters, epidemics/pandemics, or global financial crises. The literature search for this report was therefore initially limited to studies that focused on social protection measures in response to previous shock events, including Ebola, severe acute respiratory syndrome (SARS), Middle East respiratory syndrome (MERS), and Zika outbreaks, because these provide learnings that are most likely to generalize to the current COVID-19 pandemic.^7^

The literature included in this rapid scoping review was also limited to focus on materials that can specifically inform the COVID-19 *recovery* period. The decision was therefore made to exclude documents that focused primarily on the design and implementation of social protection interventions in the early response phase of shock events. This is because the purpose of this rapid scoping review is not to discuss the range of social protection measures that have been introduced in response to COVID-19. That information has already been compiled in a working paper by Gentilini et al.^8^ as well as the aforementioned scoping review by Hebbar and Phelps.^5^ Instead, this scoping review builds on these reports, with a particular focus on “what comes next” for social protection as we transition into the COVID-19 recovery period.

The search terms described below were compiled and refined based on an initial search of the literature on these topics using the Google Scholar database. This produced 2 sets of search terms, which were then used to identify materials in the PubMed and PAIS International databases. These databases were selected for searching based on the recommendation of York University librarians to capture a broad range of interdisciplinary literature. Each search was completed for each database. The search terms were carefully tailored through a process of testing and refining the terms through searching the aforementioned databases to generate a manageable number of returned documents, which were most relevant to the topic of this scoping review.

### Search 1: “Social Protection” and (Ebola or SARS or MERS or Zika or “COVID-19” or COVID or Coronavirus or “Corona Virus”)

In the PubMed database, results were limited to English-language materials published in the past 10 years. The scope was expanded from the initial search, which had been limited to the past 5 years, due to the small number of articles initially returned. This search returned 12 articles initially considered relevant to this project. After further screening, 7 were found to be relevant to this scoping review. A limitation of the PubMed database for this research topic was that results tended to be highly focused on biomedical topics.

In the PAIS International database, results were limited to English-language materials published in the past 5 years. This search returned 119 journal articles and working papers. After screening and removal of duplicate results, 15 were found to be relevant to this scoping review.

### Search 2: (“Covid-19” or “Covid” or “Corona Virus” or “Coronavirus” or “Ebola” or “SARS” OR “MERS” or “Zika”) AND (“Cash Transfer” or “School Feeding” or “School Meal” or “Food Assistance” or “Vouchers” or “Child Benefits” or “Public Works”)

In the PubMed database, results were limited to English-language materials published in the past 10 years. The scope was also expanded from the initial search, which had been limited to the past 5 years, due to the small number of articles found. This search returned 12 articles. After initial screening and removal of duplicate results, 4 were found to be relevant to this scoping review.

In the PAIS database, results were limited to English-language materials published in the past 5 years. This search returned 154 articles. After initial screening and removal of duplicate results, 6 were found to be relevant to this scoping review.

Reviewing the reference lists of papers returned through these database searches led to the identification of 17 additional articles. The author then reviewed the full text of all 49 articles identified through this process.

## The Argument for Social Protection During the COVID-19 Recovery Period

Social protection measures can serve 2 different but overlapping purposes. They can serve as a temporary *protection* for those at risk of falling below a certain threshold of well-being (as in the case of many temporally limited programs introduced to mitigate the shock associated with the COVID-19 pandemic). Alternatively, they can be focused on providing more permanent forms of security, with an aim of *promoting* improved well-being among those experiencing chronic poverty.^1,3^

As of mid-June 2020, at least 1,024 different social protection measures had been planned or implemented by 195 countries or territories around the world.^8^ This includes cash transfers (271 measures in 131 countries/territories), in-kind food or voucher schemes (116 measures in 87 countries/territories), and utility/financial obligation supports (154 measures in 93 countries), which together account for the majority of the response.^8^ School feeding programs (27 measures in 25 countries) and public works programs (14 measures in 11 countries) were less common.^8^ Cash transfer programs were by far the most widespread form of social protection to be introduced in response to the COVID-19 pandemic, although this category of measures was heterogeneous. Not only were these programs geographically widespread, but they also reached 1.2 billion of the 1.7 billion individuals around the world who have benefited from some form of social protection in response to COVID-19.^8^

Although many countries were beginning to bring COVID-19 cases under control at the time of writing, other consequences associated with the pandemic still posed “a major threat to the well-being of people and nations worldwide.”^9^^(p10)^ This means that social protection will likely continue to play a critical role in combating harms associated with COVID-19 throughout the recovery period.

The need to address “aftershocks” that ripple out from the initial shock event can be seen in a qualitative study of the nutritional consequences of the Ebola virus in Sierra Leone between 2014 and 2016. That outbreak led to ∼14,000 cases and nearly 4,000 deaths.^10^ Similar to interventions that have been recently introduced to limit the spread of COVID-19, responses to the Ebola virus in this time and place included quarantine and isolation periods, which devastated local economic productivity.^10^ In this agricultural population, impacts were not limited to food production and supply chain disruptions (although in the short term, this did lead to a nutrition crisis), but also had cascading effects upon social dynamics, the health system, and the wider economy.^10^ The point made by Kodish et al.^10^ is that this crisis and its long-term consequences were worsened by inadequate investments in social protection and by insufficient shock event preparedness. Another lesson from this case study is, however, that although social protection measures might intuitively have been concentrated on aiding the needs of farmworkers, in reality, measures that spanned across many sectors of society, and continued to provide support throughout the recovery period, were what was needed for a timely return to status quo.^10^

Turning to the present day, a recent study by Martin et al.^11^ illustrates the difference that social protection measures could make in mitigating the scale of negative impacts associated with the COVID-19 shock event. Their economic modeling simulation used the population of the San Francisco Bay Area as a case study to predict outcomes following a hypothetical 3-month lockdown period. In the absence of any social protection measures being put in place, the model predicted local poverty would increase from a baseline rate of 17.1% to 25.9%, with households taking about a year to recover financially.^11^ When state-level unemployment insurance and federal CARES stimulus benefits were introduced to the model, the poverty rate rose to only 19% and the recovery time was shortened to 6.7 months.^11^ It should be noted, however, that this model is based on a high-income country. While the population is heterogeneous, they are not representative of the experiences of individuals in low- and middle-income countries who likely have smaller (or nonexistent) savings to cushion the impact of this shock event. The authors further note that the pandemic has forced many households to access their savings, and so should these populations suffer another shock within the year or so before the recovery period is complete, the outcome is likely to be devastating.^11^

In the absence of widespread and sustained social protection measures, COVID-19 is likely to have a catastrophic impact on global poverty rates, given that as many as half a billion people are at risk of being pushed below the poverty line due to the pandemic, while between 40 and 60 million people are at risk of falling into extreme poverty.^12,13^ While the global economy may suffer US$1 trillion in losses due to the pandemic,^9^ developing countries face a “poverty tsunami,”^14^ which is likely to have intergenerational impacts on health and well-being.^9^ Although the COVID-19 shock will generate stunning effects as its impacts cascade across our complex, interconnected systems, there is opportunity in this chaos. As they hit, each wave will highlight fault lines in our systems, which will in turn signal areas to concentrate efforts to strengthen our systems during recovery.^1,15^

A robust social protection policy should therefore be balanced between measures that address pressures experienced following a shock that has already happened, those that lessen the impact of shocks that cannot be prevented, and those that build systems-wide resilience “for the prevention and reduction of shocks” that may occur in the future.^1^^(p16)^ An example of how this can be approached in the COVID-19 recovery period is offered by Norton et al.^1^ who describe linkages in policy and governance that can propel improvements in several interconnected areas to increase security via social protection (ie, social cohesion, human development, and well-being). Development in these areas reinforces effective policy and accountable governance, which in turn continues the cycle of promoting equitable growth by ensuring a productive workforce.^1^

This view of social protection policy aligns with the call to apply a *rights-based approach* rooted in the principles of universality and equity for thinking about social protection.^16^ In this light, the benefits afforded to ensure a minimum standard of living (including clothing, food, shelter, and access to basic services) are repositioned to “a right and entitlement, not just a matter of charity.”^16^^(p3)^ While the minimum standard to be tolerated would inevitably vary from context to context, the wider implication is that social protection would no longer primarily be framed as a limited-term response to shock events, but rather a responsibility of the state in the global interest of humanitarianism.^16^^(p3)^ This would effectively depoliticize social protection programs and eliminate the divisiveness that is often associated with the use of public funds for “charity” programs because citizens would recognize their universal right “to claim their [social protection] entitlements.”^16^^(p3)^

While such a proposal might sound idealist, Piron notes that the Universal Declaration of Human Rights has already established the foundation for this way of thinking.^16^ Specifically, *Article 22* (the right to social security), *Article 23* (the right to…an existence worthy of human dignity, and supplemented, if necessary, by other means of social protection), and *Article* 25 (the right to a standard to living adequate for the health and well-being of himself and of his family…[including] the right to security in the event of…circumstanced beyond his control).^17^ A commitment to ensuring social protection for vulnerable populations is thus progress toward improving all human rights.^16^ However, to participate in a social protection scheme, people need to be sure that they will not be stigmatized or otherwise penalized in the future for having done so.^4^ This can be assured through government transparency and accountability.^4^

Framing social protection as a basic right also has the ability to drive progress across several interrelated dimensions of well-being, simultaneously. For example, as a result of school closures due to the COVID-19 pandemic, more than 368 million children missed out on daily meals that were normally provided to them in this setting.^18^ These closures may therefore have significant implications for the current nutritional status of these children and may also have long-term impacts on their growth and development. Ensuring adequate access to food is not only a common goal of social protection measures in times of shock, but also a well-known strategy for improving school attendance. Increased education is a social determinant of health and well-being, and thus correlated with improved health outcomes.^3,16,19^ Actions to improve access to school-based meals in the recovery period, or those that focus on the overall nutritional status of the population or manage to keep kids in school, can all have rippling effects across each of these areas. When social protection is seen not only as a means to address crisis or “residual problems of human welfare, but as a form of policy which liberates human potential and promotes equality of opportunity as well as of outcome,”^1^^(p23)^ social protection becomes a tool that can be used to achieve transformational change.

How policymakers respond to social protection in the COVID-19 recovery period “will fundamentally shape key right-to-health questions, including how we understand government responsibilities towards health and well-being.”^15^^(p 376)^ In this respect, sociologists suggest that COVID-19 constitutes a “breaching experiment,” wherein the normal rules governing social order and the functioning of our systems have been disrupted to the point that inherent inequities are laid bare in a moment of clarity rarely seen at this scale in living history.^20^ While Gentilini et al.^8^ have highlighted the fact that the vast majority of social protection measures implemented in response to the COVID-19 pandemic have thus far been concentrated on *protection*, it could very well be that support will soon grow to refocus efforts on *promoting* well-being throughout the recovery period. On the other hand, the current pandemic has, in many cases, also had the effect of “deepening crises of social, economic, and health inequities.”^15^^(p376)^ Thus, it is also possible that we are approaching a new stage in this shock event, wherein new social protection measures may be required to address unintended harms that have followed from initial responses to COVID-19.^21^

## Equity Considerations Across Low-, Medium-, and High-Income Countries

The weight of the shock following from COVID-19 is unlikely to be shouldered equally, given that only about 10% of people in the world have had access to at least one social protection benefit.^22^ A review of Gentilini et al.^8^ efforts to compile a comprehensive list of social protection measures enacted in response to the pandemic shows that the majority of investments have been made through cash transfers in high-income countries. This is not surprising given that high-income countries have greater financial resources at their disposal; however, this concerning from an equity standpoint.

Although some middle-income countries are starting to catch up, “[t]he use of social protection as a response strategy remains nascent in low-income countries.”^5^^(p10)^ According to Renzaho,^22^^(p3448)^ investment in social protection programs in many African countries, for example, has been “very meager and insufficient.” Rather than the cash transfers and job retention programs that high-income countries have been able to invest in, African countries have been “geared towards tax payment deferrals and reductions, loans, and moratorium on debt payments, with little on offer for boosting and maintaining employment and supporting individuals and households.”^22^^(p3448)^ Given that more than three-quarters of those at risk of falling into extreme poverty as a result of COVID-19 live in sub-Saharan Africa or southern Asian countries, this is also a global equity concern.^23^

Even when social protection measures are in place, the problem remains of how to reach people who are more vulnerable because they earn their living in the undeclared economy.^9^ The undeclared economy refers to all work that generates earnings that are not declared to the government.^24^ The reasons that people may earn their income through the undeclared economy are diverse; however, it is generally held to result from necessity after individuals have been excluded, for whatever reason, from earning wages in the formal or “declared” economy.^24^ Work in the undeclared economy is more prevalent in low-income countries and represents about 61% of the global workforce.^24^

Three categories of workers fall within the undeclared economy: (1) those who are completely undeclared and have no formal work contract; (2) those who are under-declared and either have 2 sources of income but only report one, or report an income lower than that which they actually receive; and (3) those who misrepresent their self-employed status.^24^ In each of these cases, workers suffer from limited access to social protection measures, either because they do not meet the criteria to qualify for support programs or because they may receive less support relative to their usual income than they might otherwise qualify for, both of which are likely to result in a decline in their standard of living.^24^

Williams et al.^24^ suggest that social protection measures intended to respond to the COVID-19 shock might also be leveraged as a means to pull some of these individuals into the declared economy, thereby not only ensuring that benefits are extended to vulnerable individuals, but also advancing action focused on improving work situations for everyone. One way that this can be accomplished is through voluntary disclosure programs whereby people are offered clemency for reporting previously undeclared sources of income and thereby granted eligibility to receive social protection benefits under an agreement that they will continue reporting their income in the future.^24^ Voluntary disclosure programs have been proven as an effective means for improving the problems associated with work in the undeclared economy. In 2003, the United Kingdom introduced such a program. Although they wrote off £2.7million in penalties in exchange for 3,000 registrations, this translated to £11.4 million in recovered tax income and £2.5 million in fines for those who did not uphold their agreement to report future income.^24^ Overall, these efforts were found to enhance resilience within the workforce so that converts to the declared economy were better positioned to withstand the next shock event.

## Gender and Racial Inequities

Although few will avoid experiencing any negative impacts associated with the COVID-19 shock event, it is expected that the consequences will not only be disproportionate when gender, age, and race are factored in, but that the recovery period will also last longer for certain groups.^5^ Not only is vulnerable status likely to worsen a person's experience with COVID-19, but the pandemic is also likely to worsen the impoverishment of vulnerable populations.^5^ Women and their children, as well as migrants (especially refugees), have been identified as priority populations to target for social protection, given that hardships associated with this shock are expected to be unevenly experienced among these groups and to lead to further declines in their socioeconomic statuses as time goes on.^5,9,12^

It is not possible to generalize about considerations for addressing gender and racial inequities when implementing social protection measures because problems and “solutions must be context-specific.”^12^^(p3)^ Rather, thinking about this should always be local and start with “the needs, realities, and priorities of the groups which are intended to benefit.”^1^^(p15)^

As a case in point, the nutritional status of women and children worldwide is expected to significantly deteriorate as a result of the COVID-19 pandemic. Modeling suggests that wasting may increase in the range of 10% to 50%, while excess child mortality could rise to an upper-end projection of 2 million deaths in low- and middle-income subpopulations as a result of the shock.^25^ While these figures are alarming, they do not take into consideration the additional health consequences resulting from nutritional deprivation on maternal health or children's development.^25^ This is important given that the COVID-19 shock currently threatens to reverse gains on linear growth in children.^12^

A feasible solution to ameliorate this crisis might be to immediately fund a social protection program targeting support for women and children's households. Yet, such measures could have the unintended result of actually worsening domestic inequality and thus unintentionally exacerbating the situation for women and children. This is because social protection measures implemented at the household level may fail to consider how resources are distributed and controlled among individuals.^1^ In many cases, targeting social protection to households means placing resources under men's control, which can have different consequences for different members of the household.^1,4^ Financial aid aimed at household units also fails to consider that informal support networks often play an important role in women's economies.^12^ These networks are likely to be among those that have been disrupted during shock events and may be a better place for focusing interventions to support women and children during a shock than injecting capital into household economies. It is therefore important to consider intrahousehold dynamics from both gender and intergenerational lenses to ensure that interventions are appropriate and do not contribute to exacerbating existing inequalities.^1,19^ This is, in part, what Norton et al.^1^^(p16)^ are referring to when they say that it is likely that low- and middle-income countries will need to “take account of the rich variety of institutions outside the public sector which [can] provide social protection functions,” including exploring opportunities to “engage with informal, traditional, and private systems so that public policy makes best use of their potential.” This can serve to (1) increase the absolute level of resources available for social protection measures, (2) increase efficiency and reach of interventions through trusted institutions and networks, and (3) provide local-level knowledge needed to ensure programs are culturally appropriate and respond to real needs and priorities in practical and meaningful ways without widening inequities or causing unintended harms.^1,22^

Refugee groups are another priority population that may require dedicated social protection supports in the COVID-19 recovery period. Currently, 85% of refugees live in low- and middle-income countries, making their position especially precarious.^26^ Alio et al.^27^ note that economic shutdowns have pushed many previously self-sufficient refugees into extremely difficult positions. Not only do refugee groups often live in cramped quarters that can contribute to the spread of the virus, but many are at risk of falling between the cracks in local systems as they may not qualify for social protection measures, while simultaneously being disconnected from informal support networks back home that they might otherwise have turned to.^26,27^

The situation is made worse by the fact that many staff from nongovernment organizations and even the United Nations have been removed from refugee camps due to the COVID-19 threat.^26^ In some cases, these gaps are being filled by refugee-led groups, which are organizing to provide forms of social protection for fellow refugees.^26^ These groups reflect a common preference among many refugee groups to first seek community-level support in times of need.^26^ Although they are rarely funded by international aid, these groups should nevertheless be identified and considered as key partners.^26^ This position is supported by evidence from Lebanon, where local-level refugee organizations have successfully organized social protection programs to deliver food, cash, and in-kind supports for “tens of thousands of families.”^27^^(p78)^ Betts et al.^26^ note that the strength of engaging organizations at the local level is that they are often holders of knowledge and relationships necessary for reaching vulnerable populations, and they are often well-positioned to aid in mapping organizational capacity and identifying practical means for delivering resources into the hands of community-level providers.

Furthermore, evidence supports the position that engaging with local communities and partnering with local organizations to design and deliver social protection measures in response to locally identified concerns increases the likelihood that change will be sustained in the future.^28–30^ As communities have pulled together to support one another through the challenges stemming from the pandemic, the opportunity to leverage the momentum of this nature may never again be so great.

## “Greening” the Social Protection Response

While the economic and health impacts of COVID-19 have been widely commented upon in academic and gray literature, environmental concerns have thus far received very little attention. Leal Filho et al.^9^ note that their search for literature related to the pandemic returned more than 4 million results, with 68% of the first 100 articles returned focusing on economic and business implications.^9^ Yet, not a single result among these 100 articles touched upon environmental considerations.^9^ Thus, some researchers have begun to call upon decision makers to not lose sight of environmental considerations when considering the next steps in the pandemic response and recovery.^6^

While we wait for the literature specific to the COVID-19 pandemic to catch up, we can look for lessons from a previous shock event—the 2007-2008 global financial crisis—to inform recommendations for “greening the COVID-19 recovery.”^6^^(p6)^ Such efforts may find a toehold by aligning with a discourse on healthy public policy; however, this work should proceed with caution because many approaches for linking environmental concerns and population health that would have made sense a year ago may not be appropriate today. An example of this can be seen with investing in mass transit to reduce pollution and increase affordable transportation; for the time being, this is out of touch with public health concerns, which are instead favoring investments in “soft mobility” such as walking and bike riding.^6^

Given the current uncertainty, those with expertise in this area caution that as recovery plans are developed, it may very well be that the best we can hope for is a commitment to “do no harm,” thereby ensuring that we hold the line on environmental standards to not lose ground on progress made to date.^6,18^ This stance should also include oversight to ensure that any programs or standards that were loosened in the interest of immediate COVID-19 responses are fully reinstated as soon as it is safe to do so.^6^

## Building Resilience Through Action on Sustainable Development Goals

The COVID-19 pandemic has shed light on how unprepared our interconnected systems are to handle a global crisis of this scale^15,18^ and highlighted the vulnerabilities that characterize many of the world's poorest and most marginalized groups.^9,31^ While the COVID-19 shock event has been unparalleled in magnitude,^5^ recent history (ie, H1N1, SARS, MERS, and Ebola virus) unfortunately shows that we should expect and prepare for more of these kinds of shock events to occur in the future.^32^ As we consider the COVID-19 recovery, the focus on social protection should therefore be 2-fold. First, measures should be enacted to ensure that everyone, everywhere, does in fact recover from this shock, lest we lose ground on progress made to date to address poverty and other social determinants of health.^33^ Second, the recovery period should also be approached as an opportunity to strengthen our systems and “build back better.”^33,34^ The timing is opportune, as action on these fronts may be met with unusually high levels of both public support and political will, given that we are still living with the effects of our current systems having been “stress-tested” by the COVID-19 pandemic.^33^

Hebbar and Phelps^5^^(p15)^ agree that “[a] shock is an opportunity to build system resilience” by learning from changes made in response to stressors and applying these lessons to “strengthen the entire system for the future.” Thus, although we are likely to remain in a period of uncertainty throughout the COVID-19 recovery, and we may therefore need to adjust responses along the way, we can nevertheless be sure about where the fault lines in our systems are and prioritize addressing those as we move forward.^9,35,36^ There is precedent for the kind of agile responses these researchers are calling for. The SARS epidemic, for example, highlighted weakness in China's public health system.^37^ As a result, significant public health reforms were implemented during the recovery period, including the New Cooperative Medical Scheme, which introduced affordable health insurance and had a range of positive impacts for rural populations.^37^

While social protection measures have been widely used in the past, this has most often been in direct response to infectious diseases, natural disasters, or economic crises.^5^ Building up to a level of resilience that could see an end to the need for “just-in-time” funding for social protection measures could, however, significantly reduce the impact of future shocks, serve as an “automatic stabilizer,” and thereby lessen the amount of investment needed to respond to shocks when they occur.^33^^(p11)^ Framing social protection in this way also opens the door to leveraging investment in this area for transformational change.^2,4,38^

The Agenda 2030 sustainable development goals approved by the United Nations in 2015 include 169 objectives under 17 broader goals based on the principle that economic growth can only be sustainable alongside action to reduce inequity and ensure environmental sustainability.^39^ Many of these goals directly intersect with social protection measures introduced during the COVID-19 response, and many will be impacted by actions that occur in the recovery period. Investing in efforts to reduce vulnerability through social protection measures can not only reduce immediate human suffering resulting from COVID-19, but building on these efforts will also build resilience for future shocks and reduce global inequities by way of intervening on a variety of social determinants of health.^40^

This is important because a key reason for the inequities that we are witnessing associated with the COVID-19 shock are symptomatic of “economic trends that have already been shaping well-being for many years before the COVID-19 pandemic,” which, as it happens, the sustainable development goals were intended to address.^34^^(p3)^ Thus, one approach for leveraging social protection programs introduced for the COVID-19 response for transformational change (via progress on sustainable development goals) is to begin thinking about the benefits that social protection programs have been providing as minimum standards to which all people are inherently entitled.^1^ Unless the weaknesses in our systems highlighted by the COVID-19 shock are addressed at their roots, social protection measures will always be a bandage measure that mitigates symptoms, not causes, and, in the long term, is ultimately unsustainable.

While some are pessimistic about whether it will still be possible to meet the timeline for achieving the sustainable development goals, given that progress was already lagging prior to COVID-19, others are cautiously optimistic that vulnerabilities exposed (or once more brought to the forefront) by this shock event may be a catalyst for a renewed commitment to meeting these targets:

Indeed, the global crises triggered by COVID-19 mean that pursuing and implementing [the Sustainable Development Goals] are more important now than they were before, since they represent some of the means via which quality of life can be restored and the many problems associated with lack of water, food, or poor health conditions may be addressed. In doing so, the momentum created by the pandemic may lead to a transformation from what currently is regarded as a global threat, to a global opportunity, providing a new impulse leading to the realization of the UN Agenda 2030 as a whole, and of the [Sustainable Development Goals] in particular.^9^^(p10)^

The COVID-19 shock has even shown us that it might be time to shift thinking on the sustainable development goals, and reposition the third goal, of achieving good health and well-being, “at the center of the Agenda.”^19^^(p2)^ This is because the pandemic has once more reminded us that all other goals are ultimately dependent upon this one. What follows is a theoretical model for recasting social protection measures already implemented in response to the COVID-19 shock as opportunities for achieving the kind of transformational change that will be required to make progress on the sustainable development goals in the COVID-19 recovery period in general, but on goal 3 in particular.

While responses to the COVID-19 shock have concentrated on protective measures, there is opportunity inherent to the way in which social protection is conceptualized as a continuum (ranging from protective to preventative, to promotional, to transformational programs and policies), because such a view implies the innate ability to build up, scale, and spread proven responses over time.^2^ Another advantage of this model is that it offers a common approach for countries that may be starting at different times, or that may have different levels of resources to contribute (and thus may progress along this scale at different paces), to nevertheless share a common path for building on social protection measures to achieve results in line with sustainable development goals that they have already agreed to drive toward.^1^

An example of how this can look in practice can be seen in the use of “graduated” cash transfers to not only mitigate the immediate pressures of living in poverty, but also to serve as means for systematically lifting people out of poverty. The example of cash transfers is ideal, given how widespread this social projection measure has been during the initial response to the COVID-19 shock.

While cash transfers are often introduced for a limited period of time to help offset the pressures immediately following a shock event, Devereux and Sabates-Wheeler^41^ have argued for building up a graduated program around cash transfer measures to help individuals climb out of extreme poverty. Graduated cash transfer programs provide a packaged series of steps that go beyond the initial cash transfers to help equip recipients for earning higher incomes and improved livelihoods.^41^ These programs are rooted in a theory of change wherein households experiencing extreme poverty qualify to receive regular cash transfer payments for a period of 1 to 2 years.^41^ Typically, the payments are supplemented early on with asset transfers (such as livestock), which can serve as a future income source when the cash transfers end.^41^ The predictability of the payment schedule combined with the injection of other assets enables families to start putting some money away into savings, and soon many also qualify for microcredit financing or small loans that can be used to start new businesses.^41^ Throughout this process, “training in income-generating activities plus coaching in life-skills” is offered, to build financial literacy and set people up for success when they “graduate” from the program.^41^^(p2)^ The key to the success of graduated cash transfer programs is its holistic nature, which functions to “(1) stabilize household consumption, (2) protect assets against being sold to meet basic needs, and (3) relieve liquidity constraints, allowing households to make productive investments.”^41^^(p2)^ The impact of graduated cash transfer programs in places such as Bangladesh and Rwanda has been described as “astonishing” due to the outcomes they generate through transforming lives and livelihoods.^41^^(p2)^

This example highlights the potential to leverage social protection programs to achieve transformational change, and also serves as an example of how social protection measures implemented in response to a shock event could be scaled and spread to make progress toward sustainable development goals. Gradated cash transfer programs clearly align with goal 1, which is to *end poverty in all its forms everywhere*, and, in particular, target 1.3, which calls for *implement[ing] nationally appropriate social protection systems and measures for all, including floors, and by 2030 achieve substantial coverage of the poor and the vulnerable*.^39^

The popularity of cash transfer programs among social protection responses to COVID-19 is not surprising given that they have been widely demonstrated as an effective means for addressing immediate poverty.^42^ Cash transfers programs are based on the premise that “the key constraint for poor people is simply lack of money, not knowledge, and thus they are best equipped to decide what to do” to address their immediate situation and long-term standard of living.^43^^(p10)^ This also mitigates risks inherent when social protection programs must be designed and implemented quickly, without extensive knowledge of the local context. Rather than making assumptions about what is needed or what will work “best” for recipients, policymakers can leave this decision up to those who know what their immediate needs are and how they can best be met. The reliable stream of income that cash transfers provide has not only been found to reduce the proximate pressures of living in poverty, but can also increase participation in a range of education and public health programs when these are identified conditions of payment.^44,45^

Any progress made on sustainable development goals during the COVID-19 recovery period would be cause for celebration, but care should also be taken to evaluate the implications this could have for potentially widening global equities. Low-income countries, which are now also struggling to finance social protection measures in response to the pandemic, are much less likely to have the capacity to also move the needle on sustainable development goals.^9^ Throughout the COVID-19 recovery, therefore, there “needs to be more international solidarity, in the form of a great political commitment across all nations”^9^^(p10)^; increased international cooperation^34^; and improved coordination among global, regional, and local levels,^18^ so that developing countries do not emerge from the COVID-19 shock further disenfranchised within the global community.

## Limitations of the Evidence and Gaps to be Addressed Through Future Research

Agrawala et al.^6^^(p6)^ observed a “remarkable death” of evaluation evidence that could speak to outcomes attributable to social protection programs in general, but especially with regard to recovery measures introduced in response to previous shock events. Consequently, they have highlighted “the need for systematically building in evaluation frameworks” into social protection responses.^6^^(p6)^ Similarly, Hillier-Brown et al.^46^^(p2)^ wrote that the “systematic review evidence-based on the effects of social protection policy interventions remains sparse, of low quality, of limited generalizability…and relatively inconclusive.” Because “little in the way of impact evaluation has taken place,” we are still at a very early stage of being able to agree on fundamental qualities of “strong” versus “weak” social protection policies.^47^^(p2)^ Furthermore, in light of the humanitarian implications of social protection measures, cost–benefit comparisons are neither sufficient nor appropriate for arguing for which social protection measures have been “better” or “worse,” because “their utility cannot be objectively determined.”^42^^(p2)^ Thus, despite how much of our collective health and economic well-being currently rests upon social protection measures, we find ourselves in the unenviable position of having good intentions, but little evidence to support the merit that we intuitively expect will follow from investing in social protection programs.

The reason for this is that although there is a great deal of literature available, contextualization is paramount.^22^ Each article on the topic of social protection differs widely by target population, circumstance, local needs and priorities, measures implemented, and the duration of the intervention. Thus, while there is considerable evidence to suggest that social protection measures can be effective, “many questions remain about how such programs should be designed, implemented, and tailored to local conditions.”^3^^(p161)^ This represents the most significant gap in knowledge in the field of social projection literature. Fortunately, not only can this gap be addressed through implementation science research,^18^ but a peer-reviewed study was recently published that specifically aimed to answer the question of how research should be planned to do so.

Qui et al.^3^ mixed methods study combined insights from a review of social protection literature, interviews with policymakers from low- and middle-income countries, and researchers from around the world to identify and then rank research priorities for informing evidence-based approaches to implementing social protection programs with a mind to advancing action on the sustainable development goals. The top 10 results (ranked most to least important) are as follows:

1. How can social protection programs for health be designed, implemented, and evaluated to ensure sustainability and scalability in low- and middle-income countries, including conflict-affected settings?
2. What are the contextual factors that influence the effectiveness of conditional and unconditional cash transfer schemes for health?
3. How can various social protection initiatives be best integrated or harmonized across sectors?
4. How cost-effective are conditional cash transfer programs compared to supply-side interventions (eg, strengthening the quality of infrastructure and expanding services) in improving health?
5. What are the impacts of social protection programs in conflict-affected settings and their effectiveness in improving health outcomes and access to health services?
6. What are the pathways through which social protection programs affect clinical and nonclinical outcomes, and what are the implications on program design?
7. What is the impact of social protection initiatives on health equity outcomes and equitable access to quality health care services for poor and marginalized populations?
8. How can routine information systems be strengthened and used to monitor and evaluate social protection systems for health?
9. How do social protection programs influence the interaction between public and private health care providers with regard to service availability, quality of care, and utilization?
10. What are the effects of unconditional cash transfer programs on health care quality, coverage, and outcomes across settings in low- and middle-income countries?^3^^(p164)^

A common thread in the literature reviewed for this report is the difficulty that decision makers will ultimately face in committing to evidence-based decision making because so little information about outcomes is currently available. While neither a “strength” nor a “limitation” of the extant literature, it should be noted that when contextualized information does become available, care should be taken to ensure that that information is carefully and frequently (re)evaluated because the landscape will continue to rapidly evolve in response to pressures and actions following from the initial COVID-19 shock.^6^

 Building evaluation frameworks into social protection responses throughout the recovery period should be prioritized so that evidence will be available to inform future efforts.^34,42^

This will require a commitment of both time and resources, given that evaluation projects typically require sustained investment for months or even years after a program concludes to complete data collection, analysis, and knowledge translation. This may add considerable costs to the overall budget for a social protection intervention, which in turn represents an ethical dilemma, as the decision to invest in evaluation may require diverting funds away from potential beneficiaries.^42^ This is a trade-off that will need to be weighed by decision makers; however, the evidence generated through a well-executed evaluation plan is likely to contribute significant long-term value.

 The following measures proposed by Norton et al.^1^ offer a strong starting point for designing an evaluation framework at the local level:

- Responsive: to the needs, realities, and conditions of livelihood of those who are intended to benefit
- Affordable: both in the context of short- and medium-term planning for the public budget and in terms of not placing unreasonable burdens on households and communities
- Sustainable: both financially and politically, with a requirement on government to ensure that the state's role in social protection reflects an adequate level of public support for interventions to assist the poorest
- Adopted: institutionally, with sustainable structures of governance and implementation whether in the state or civil society structures.
- Inclusive: built on a principle of utilizing the capabilities of individuals, households, and communities and avoiding the creation of dependency and stigma.
- Flexible: capable of responding to rapidly changing scenarios and emergence of new challenges…and of meeting the changing needs of individuals within the lifecycle.^1^^(p10)^

In addition, Hebbar and Phelps^5^ offer these questions, which could be used as a starting point for an evaluation of global responses:

- How successful have countries been in efforts to “reconfigure the range and scale of enrolment channels”?
- How quickly were governments able to “ramp up capacity to meet the surge in demand for social protection”?
- How responsive were “administrative processes to address increased demand while ensuring timely response”?^5^^(p13)^

Investing in the evaluation of social protection measures introduced to address the COVID-19 shock will allow us to better understand and predict consequences of program and policy interventions in the future.^34^ This is critical because the current pandemic has reinforced what we already knew; the systems that we rely on in the best of times, as well as in times of disruption, are increasingly “susceptible to widespread, irreversible, and cascading failure.”^34^^(p1)^

## Conclusions

Koehler^4^ and Plamondon et al.^48^ warn that although well-planned social protection measures are widely held as a positive approach, they are nevertheless aimed at ameliorating the symptoms of the underlying problems, which ultimately lurk in the flawed systems that we have created for ourselves. This is not to say that investments should not be made in social protection, but rather that attention must also be directed at understanding the systematic underpinnings of the inequities that necessitated social protection measures in the first place.

When the human suffering attributed to the COVID-19 shock is framed as an injustice, rather than simply as misfortune, there will be an opportunity to work toward addressing the root causes of inequity, which will equate to both stronger systems and stronger people. Shock events such as the current COVID-19 pandemic “provide limited windows of opportunity for effecting large changes in the system. Indeed, when major changes in human rights practices occur, it is often because of such an event.”^49^^(pp2003–2004)^ By exposing inequities inherent in our systems, the pandemic has revealed a way forward in the recovery period.^15^ The opportunity to leverage the disruption caused by the pandemic to effect a large-scale change as we enter the recovery period is uncharted, but it is not unprecedented.
